# Integrating the serum proteomic and fecal metaproteomic to analyze the impacts of overweight/obesity on IBD: a pilot investigation

**DOI:** 10.1186/s12014-023-09396-y

**Published:** 2023-02-09

**Authors:** Ping Yan, Yang Sun, Juan Luo, Xiaolin Liu, Jing Wu, Yinglei Miao

**Affiliations:** 1 Kunming Medical University, Kunming, China; 2 Department of Gastroenterology, First Affiliated Hospital of Dali University, Dali, China; 3 Department of Gastroenterology, First Affiliated Hospital of Kunming Medical University, Kunming, China; 4 Yunnan Province Clinical Research Center for Digestive Diseases, Kunming, China

**Keywords:** Inflammatory bowel disease, Ulcerative colitis, Crohn’s disease, Overweight and obesity, Proteomic, Metaproteomic, Gut microbiota

## Abstract

**Background:**

Inflammatory bowel disease (IBD) encompasses a group of chronic relapsing disorders which include ulcerative colitis (UC) and Crohn’s disease (CD). The incidences of IBD and overweight/obesity are increasing in parallel. Here, we investigated alterations in proteomic in serum and metaproteomic in feces of IBD patients with overweight/obesity and aimed to explore the effect of overweight/ obesity on IBD and the underlying mechanism.

**Methods:**

This prospective observational study (n = 64) comprised 26 health control subjects (HC, 13 with overweight/obesity) and 38 IBD patients (19 with overweight/obesity) at a tertiary hospital. Overweight/obesity was evaluated by body mass index (BMI) and defined as a BMI greater than 24 kg/m^2^. The comprehensive serum proteomic and fecal metaproteomic analyses were conducted by ultra-performance liquid chromatography-Orbitrap Exploris 480 mass spectrometry.

**Results:**

UC and CD presented similar serum molecular profiles but distinct gut microbiota. UC and CD serum exhibited higher levels of cytoskeleton organization- associated and inflammatory response-related proteins than the HC serum. Compared the serum proteome of UC and CD without overweight/obesity, inflammatory response-associated proteins were dramatically decreased in UC and CD with overweight/obesity. Fecal metaproteome identified 66 species in the feces. Among them, *Parasutterella excrementihominis* was increased in CD compared with that in HC. UC group had a significant enrichment of *Moniliophthora roreri*, but had dramatically decreased abundances of *Alistipes indistinctus*, *Clostridium methylpentosum*, *Bacteroides vulgatus,* and *Schizochytrium aggregatum*. In addition, overweight/obesity could improve the microbial diversity of UC. Specifically, the UC patients with overweight/obesity had increased abundance of some probiotics in contrast to those without overweight/obesity, including *Parabacteroides distasonis*, *Alistipes indistincus*, and *Ruminococcus bromii*.

**Conclusion:**

This study provided high-quality multi-omics data of IBD serum and fecal samples, which enabled deciphering the molecular bases of clinical phenotypes of IBD, revealing the impacts of microbiota on IBD, and emphasizing the important role of overweight/obesity in IBD.

## Introduction

Inflammatory bowel disease (IBD) encompasses a group of chronic relapsing disorders which include ulcerative colitis (UC) and Crohn’s disease (CD) [1]. The prevalence of IBD globally might exceed 0.3%, with approximately 7 million people affected [2]. In China, 350,000 IBD cases were registered between 2005 and 2014, and 1.5 million patients are expected to be affected by 2025 [3]. In addition to suffering from recurrent abdominal pain and diarrhea, patients have to incur high treatment costs, which limit their quality of life [4].

In some pioneer studies, investigators have found that weight loss is the common symptom presented in IBD patients [5]. Especially, patients suffering from severe IBD might lose up to 18%-62% of their weight in UC and 65%-76% in CD [6]. Contrary to the conventional perception, the increasing incidences of IBD are in parallel with prevalence rising in overweight and obesity. About 15% to 40% of IBD patients are estimated to be obese in contemporary cohorts [7]. Some researchers have reported that IBD can be complicated by obesity through increasing hospitalization rates, perianal complications, and delay in first surgery [8–10]. Thus, weight loss might serve as an obvious approach in the intervention of obese IBD patients [11]. However, whether weight loss is beneficial to the prognosis of patients with obese IBD remains unclear, and the favorable effectiveness and safety of weight-loss interventions for this population are unknown. Furthermore, other studies have found conflicting results, suggesting that high body mass index (BMI) correlates with better prognoses[12, 13], like reduced disease severity, less biologics treatment required, fewer surgery procedures and fewer hospitalization [14, 15]. Thus, a clearer understanding of the interaction between obesity and IBD becomes even more important.

Proteome and metaproteome analyses, as parts of the multi-omics approaches, provide in-depth and mechanistic insight into IBD [16, 17]. So far, studies using proteomic and metaproteome analyses have provided novel insights into the potential pathogenic mechanisms of IBD [18, 19]. Daniela et al*.* have reported that proteomic analysis of stool using matrix-assisted laser desorption/ionization time-of-flight mass spectrometry would be a novel method for IBD diagnosis and for elucidating the disease pathogenesis [20]. Another study also highlighted that an analysis of serum proteomics at diagnosis could be utilized to predict future outcome of IBD [21]. Several studies have also demonstrated that IBD is closely related to the gut microbial community dysbiosis [22–25]. Thus, gut microbial metaproteome analysis has also been applied to IBD prevention and therapeutic target discovery [26, 27]. Nevertheless, to the best of our knowledge, investigations regarding overweight/obesity influencing IBD using proteomic and metaproteomic applications are very limited.

Thus, we conducted this study to map the protein expression patterns of serum and gut microbiome in IBD patients with overweight/obesity and determine the potential molecular basis for overweight/obesity influencing IBD. We performed the serum profiling using nano-liquid chromatography coupled with Orbitrap tandem mass spectrometry (nLC-MS/MS). Orbitrap MS/MS detection was also performed for fecal metaproteome [28].

## Materials and methods

### Study participants

Between May 2021 and June 2022, a final total of 64 participants including 38 IBD patients (16 Female, 22 Male) and 26 controls (6 Female, 20 Male) were consecutively enrolled in the First Hospital of Kunming Medical University. The diagnosis of IBD patients was confirmed according to the 2018 Chinese Consensus on Diagnosis and Treatment of IBD. All IBD patients aged between 18 and 71 years with at least three years IBD history were prospectively recruited during our study period. During the same period, the health control subjects (HC) were enrolled from medical checkup recipients in our hospital, and none of them had a history of intestinal disease or symptoms. BMI ≥ 24 was used to define overweight/obesity. This study excluded patients with cancer or infectious diseases, individuals who had used antibiotics or probiotics within four weeks prior to enrollment, as well as those who had undergone intestinal surgery prior to the study. The Medical Ethical Inspection committee of the First Affiliated Hospital of Kunming Medical University approved the present study, and all participants provided informed consent in writing.

### Sample collection and protein extraction

The peripheral venous blood (3–5 mL) of each subject was drawn using anticoagulant-free tube, and the serum samples were collected after centrifugation (around 3500 rpm) for 10 min. Approximately 150 mg of fresh fecal sample was collected in a germ-free stool container. Both serum and fecal samples were immediately transferred to a − 80 °C refrigerator after collection until extraction. A total of 64 serum samples (38 IBD, 26 HC) were collected for proteomic analysis, and 64 matched fecal samples were obtained for metaproteomic profiling.

For serum protein extraction, thawed serum samples were used to remove top 14 high-abundance proteins by using Pierce^™^ Top 14 Abundant Protein Depletion Spin Columns Kit (Thermo Fisher Scientific, MA, United States), and the protein solution was then collected. Finally, the protein concentration was determined with bicinchoninic acid (BCA) assay kit (Thermo Fisher Scientific, MA, United States) according to the instructions of the manufacturer.

For fecal protein extraction, proteins from thawed feces were mechanically extracted with 500 μL phosphate buffered saline (PBS, composed of: 1.05 mM KH_2_PO_4_, 2.96 mM Na_2_HPO_4_, 155.17 mM NaCl, at pH7.4, Sigma-Aldrich). After centrifugation (15,000 g at 4 °C for 5 min), the supernatant was discarded and, the precipitate was sonicated three times on ice using a high intensity ultrasonic processor (Scientz) in lysis buffer (including 1% TritonX-100 and 1% protease inhibitor). The remaining debris was removed by centrifugation at 12,000 g at 4 °C for 10 min. Finally, the supernatant was collected and the protein concentration was determined by the BCA kit (Thermo Fisher Scientific, MA, United States).

### Protein profile analysis of serum sample

Nano-liquid chromatography coupled with Orbitrap tandem mass spectrometry (nLC-MS/MS) was applied in this study to quantify dynamic changes in the serum proteome. A following general work flow is based on the previous report by Wang et al*.* [29], which entails trypsin digestion, tandem mass tag (TMT) labeling, LC–MS/MS analysis, database searching, and bioinformatic analyses. In brief, trypsin was added at 1:50 trypsin-to-protein mass ratio for the first digestion overnight and 1:100 trypsin-to-protein mass ratio for a second digestion for 4 h. The TMT reagent kit (ThermoFisher Scientific) was then used to label digested peptides and all labeled products were desalted with Strata X C18 SPE column (Phenomenex) and dried by vacuum centrifugation. An EASY-nLC 1200 ultra-performance liquid chromatography system (ThermoFisher Scientific) was employed to separate the tryptic peptides with a linear gradient of buffers from 6 to 31% over 90 min. Buffer A was 0.1% formic acid (FA), and buffer B was 0.1% formic acid and 90% acetonitrile (ACN). The gradient was as follows: 0–68 min, 6% ~ 23% B; 68–82 min, 23% ~ 32% B; 82–86 min, 32% ~ 80% B; 86–90 min, 80% B. EASY-nLC 1200 was run at a constant flow of 500 nL/min. The separated peptides were subjected to NSI ion source Q ExactiveTM HF-X (ThermoFisher Scientific) with a nano-electrospray ion source, and 2.1 kV electrospray voltage was applied. An Orbitrap Exploris^™^ 480 MS detector was applied to analyze the separated peptides. The primary MS scan was acquired within the range of 400–1200 mass-to-charge ratios (m/z) and the scan resolution was set at 60,000. Subsequently, a normalized collision energy of 27% was set for selected peptides under secondary MS/MS analysis, with a scan range of 100 m/z and a resolution of 30,000. Automatic gain control was set at 1E5, with an intensity threshold of 5E4. A data-dependent acquisition was used for data acquisition with a dynamic exclusion of 20 s. For database searching, preliminary data were analyzed using Proteome Discoverer search engine (v2.4.1.15, Thermo) and MS/MS searching was searched against the Homo_sapiens_9606_PR_20210721.fasta (78,120 entries) concatenated with reverse decoy database with peptide false discovery rate (FDR) ≤ 0.01.

In the subsequent analysis, only quantifiable proteins (defined as proteins with a minimum of two unique peptides) were considered. A total of 1941 proteins were identified in the serum samples. The repeatability of sample replicates was estimated by Pearson’s correlation coefficient. Pair-wise replicates of samples was found to exhibit significant positive correlations. An online available PTM-BIO Shiny Tool was used to identify the differentially expressed proteins (DEPs) between IBD and HC. The identified proteins with quantitative ratios > 1.5 were considered to be up-regulated (*P* < 0.05), while proteins with quantitative ratios < 2/3 were considered down-regulated (*P* < 0.05). UniProt-GOA [30] was employed to perform gene ontology (GO) annotation of the DEPs. The Kyoto Encyclopedia of Genes and Genomes (KEGG) pathway database was applied to annotate protein pathways involved in DEPs. Metascape webtool [31] was also used to visualize the results of GO terms and pathways for DEPs between IBD with- and without- overweight/obesity.

### Metaproteome analysis of fecal sample

The extracted fecal proteins (n = 64) were subjected to fecal metaproteome analysis by trypsin digestion, TMT labeling, and LC–MS/MS analysis, as described in the section above. For database searching, preliminary data were analyzed using Maxquant search engine (v1.6.15.0) and MS/MS searching was searched against the sample_specific_database.fasta (47,024 entries) concatenated with reverse decoy database with peptide false discovery rate (FDR) ≤ 0.01.

A total of 3764 unique peptides were obtained from fecal samples. All these peptides were placed into Unipept (v2.2.1) [32] for microbial taxonomic analysis with the lowest common ancestor algorithm. Relative abundance of a species was calculated based on the intensities of identified peptides, and then species microbial composition, species diversity, and differential species analyses were conducted. Bar plots of the gut microbial community were generated to exhibit bacterial taxonomic analysis at the phylum, class, order, family, and genus levels. Alpha diversity was assessed by the Shannon index, and principal coordinate analysis was used to determine beta diversity among samples [33]. The Spearman correlation analysis was performed to determine the relationship between the human-derived proteins and calprotectin. The linear discriminant analysis (LDA) effect size (LEfSe) version 1.0 was conducted to identify differentially abundant microbiol community members among groups [34]. An absolute log10 LDA score > 2.0 was set as a cutoff criterion.

### Statistical analysis

R (version 4.0.0, R Foundation for Statistical Computing) software was used for data statistical computing and plot analysis. Two-sided *t*-test was used for the data analyses of serum and fecal samples. Details of each test are exhibited in the figures. Significances were defined as *P* < 0.05(*).

## Results

### Background characteristics of study cohorts

**Fig. 1:**
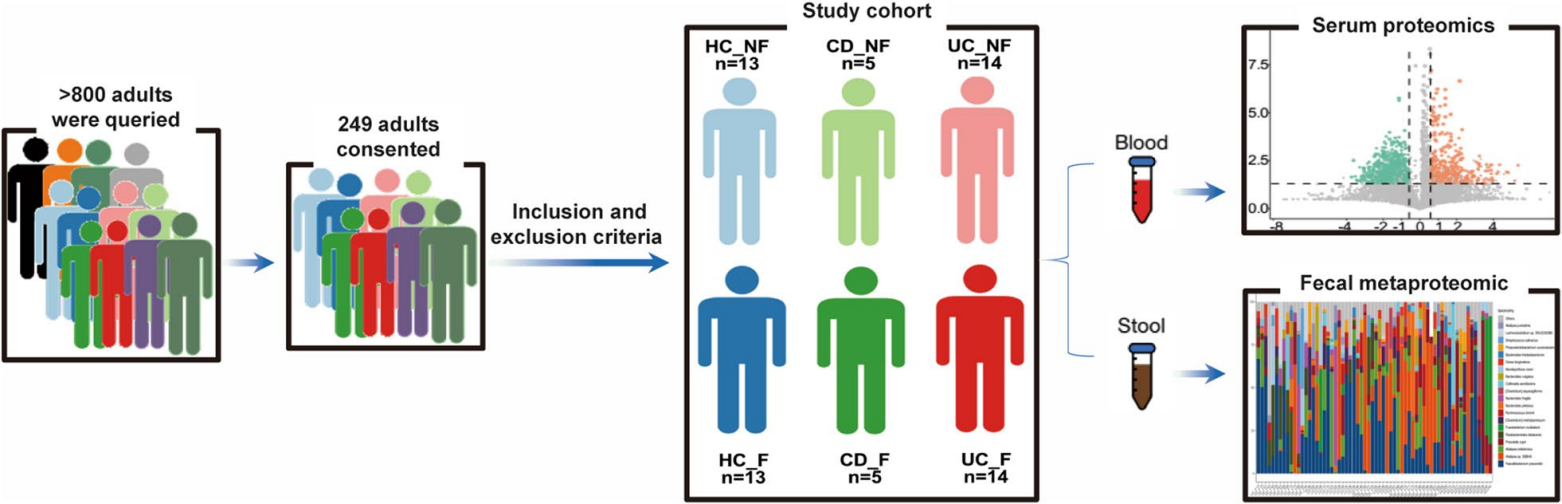
Workflow of the human experiments. Control subject, HC; Crohn's disease, CD; Ulcerative colitis, UC. For ease of representation, we here denote “with-overweight/obesity” by “F” and “without-overweight/obesity” by “NF”. Six subgroups were exhibited as UC_F, UC_NF, CD_F, CD_NF, HC_F, and HC_NF

For the entire study, more than 800 adults who attended our hospital were queried, and 249 of these queried consented to participate this study and were further screened according to the inclusion and exclusion criteria. A total of 64 participants were finally included in this study (Fig. 1). Of the 64 participants, 38 were the patients with IBD (including 28 UC and 10 CD) and 26 were control subjects (HC). Baseline data such as age, male gender, and BMI were comparable between IBD and HC groups (all *P* > 0.05, Table 1). Subsequently, 64 subjects were divided based on with- or without- overweight/obesity into six subgroups. For ease of representation, we here denote “with-overweight/obesity” by “F” and “without-overweight/obesity’’ by “NF”. Six subgroups are exhibited in Table 1 as UC_F (n = 14), UC_NF (n = 14), CD_F (n = 5), CD_NF (n = 5), HC_F (n = 13), and HC_NF (n = 13). Statistical significances were found for BMI among UC_F and UC_NF (*P* < 0.0001), CD_F and CD_NF (*P* = 0.0079), and HC_F and HC_NF (*P* < 0.0001). There was no significant difference by duration of disease between UC_F and UC_NF groups, and CD_F and CD_NF (all *P* > 0.05).

**Table 1:** Basic characteristics of inflammatory bowel disease (IBD) and controls subjects (HC)

	IBD					HC		
	Total (n = 38)	UC (n = 28)		CD (n = 10)		Total (n = 26)	HC_F (n = 13)	HC_NF (n = 13)
		UC_F (n = 14)	UC_NF (n = 14)	CD_F (n = 5)	CD_NF (n = 5)			
Age, Median (range)	43.0(18–71)	46.5(27–71)	44.0(22–62)	40.0(18–52)	30.0(27–53)	42.5(27–54)	45.0(27–53)	41.0(29–54)
Male, n (%)	22(31.8)	6(42.9)	9(64.3)	4(80.0)	3(60.0)	20(32.0)	12(92.3)	8(61.5)
BMI, Median (range)	24.36(16.16–33.22)	26.44(25.61–30.30)^a^	20.32(16.71–23.53)	27.28(25.18–33.22)^b^	18.37(16.16–19.59)	24.44(18.61–31.77)	25.38(25.00–31.77)^c^	21.36(18.61–23.88)
Duration of disease, Median year (range)	5.0 (3–7)	4.5 (3–7)	5.0 (4–7)	5.0 (3–5)	5.0 (4–6)			
Disease extent: UC, n								
Proctitis	–	0	0	–	–	–	–	–
Left-sided colitis	–	5	3	–	–	–	–	–
Pancolitis	–	9	11	–	–	–	–	–
Disease extent: CD, n								
Ileal	–	–	–	1	2	–	–	–
Colonic	–	–	–	1	1	–	–	–
Ileocolonic	–	–	–	3	2	–	–	–
Current IBD medications, n								
5-ASA	-	14	14	0	0	–	–	–
Corticosteroids	–	3	5	0	0	–	–	–
Immunomodulators	–	0	0	2	1	–	–	–
Biologics	–	0	0	5	5	–	–	–

*IBD* inflammatory bowel disease, *HC* Controls, *UC* ulcerative colitis, *CD* Crohn’s disease, *F* with-overweight/obesity, *NF* without-overweight/obesity, *BMI* body mass index, *HBI* Harvey Bradshaw index, *5-ASA* 5-aminosalicylic acid

^a^*P* < 0.0001 indicates between UC_F and UC_NF

^b^*P* = 0.0079 indicates between CD_F and CD_NF

^c^*P* < 0.0001 indicates between HC_F and HC_NF

### Proteomic characteristics of serum and metaproteomic profiles of the gut microbiome of IBD patients and control subjects

**Fig. 2:**
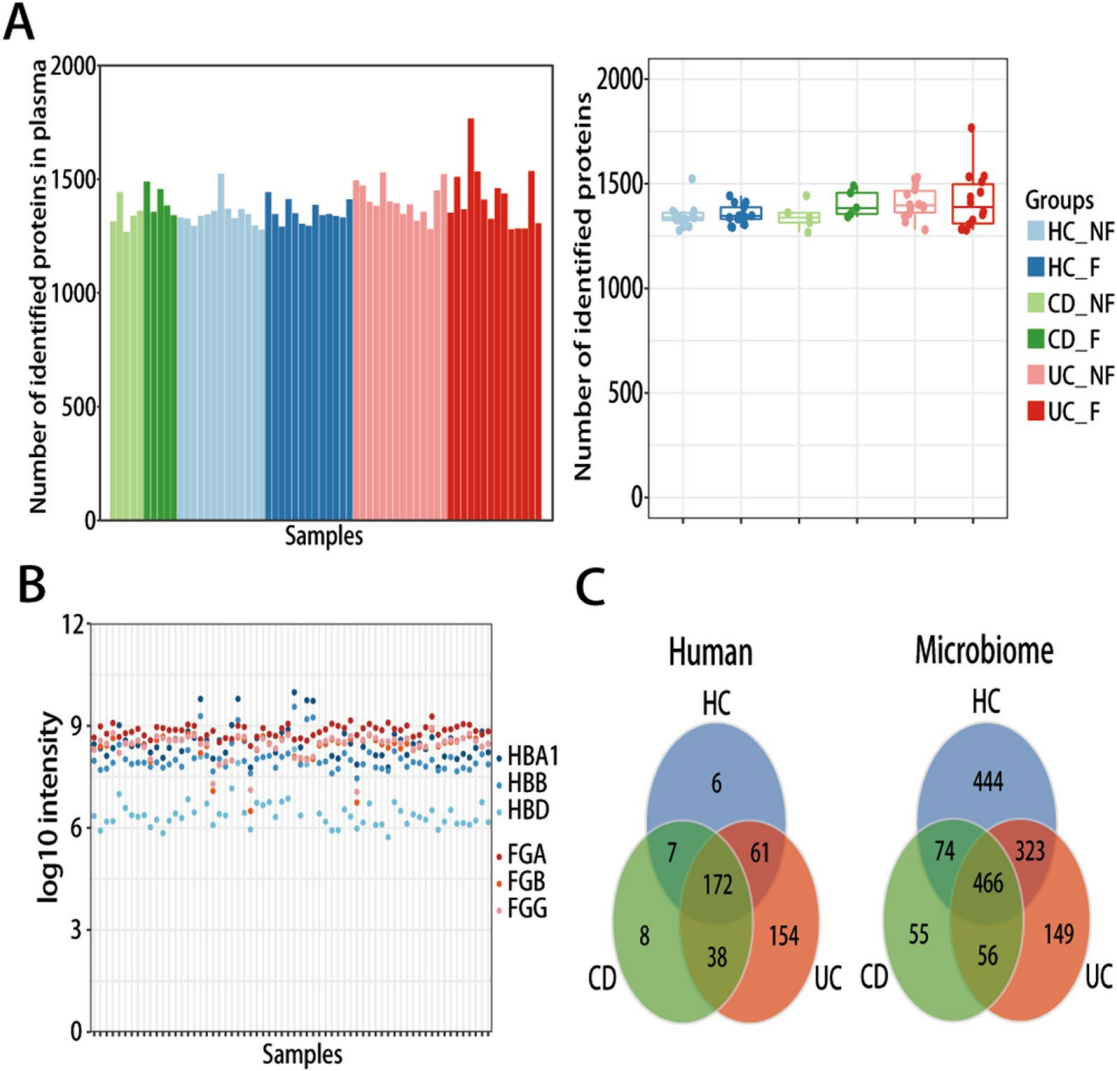
Quality control of the multi-omics data. **A** Bar-plots and boxplots showing the number of quantified proteins in serum sample among six groups. **B** Assessment of serum proteome quality by analyzing erythrocyte-specific proteins (red circles) and coagulation markers (blue circles). HBA, HBB, HBD: hemoglobin subunits alpha, beta, delta; FGA, FGB, FGG: fibrinogen chains alpha, beta, gamma. **C** Venn plots showing the identification of the human-derived and microbiome-derived proteins among HC, CD, and UC in the fecal samples

Serum proteomics experiments were conducted using nLC-MS/MS. We quantified 1350 unique proteins per subjects on average, and no significant difference was observed for identified proteins among the six subgroups (Fig. 2A). To the best of our knowledge, current proteomic depth is on par with or exceeds many other reports and is sufficient to cover most serum proteins. A published quality marker panel for erythrocyte, including hemoglobin subunit alpha, beta, and delta (HBA1, HBB, HBD), and for coagulation, including fibrinogen chain alpha, beta, and gamma (FGA, FGB, FGG), was used to evaluate the quality of the serum samples in terms of consistency of collection and handling [35]. According to the total protein profiles, no outliers were noted among samples (Fig. 2B). These findings indicate that high-quality serum samples were obtained, and the changes in serum proteins should be caused by pathological disturbances associated with the disease.

For fecal samples, 2013 identified proteins were subjected to a further analysis, including 446 (22.2%) human-derived proteins and 1567 (77.8%) gut microbiome-derived proteins, which indicates a robust quality of this fecal metaproteomic profiling (Fig. 2C).

### Serum protein alterations between IBD patients and control crowds

**Fig. 3:**
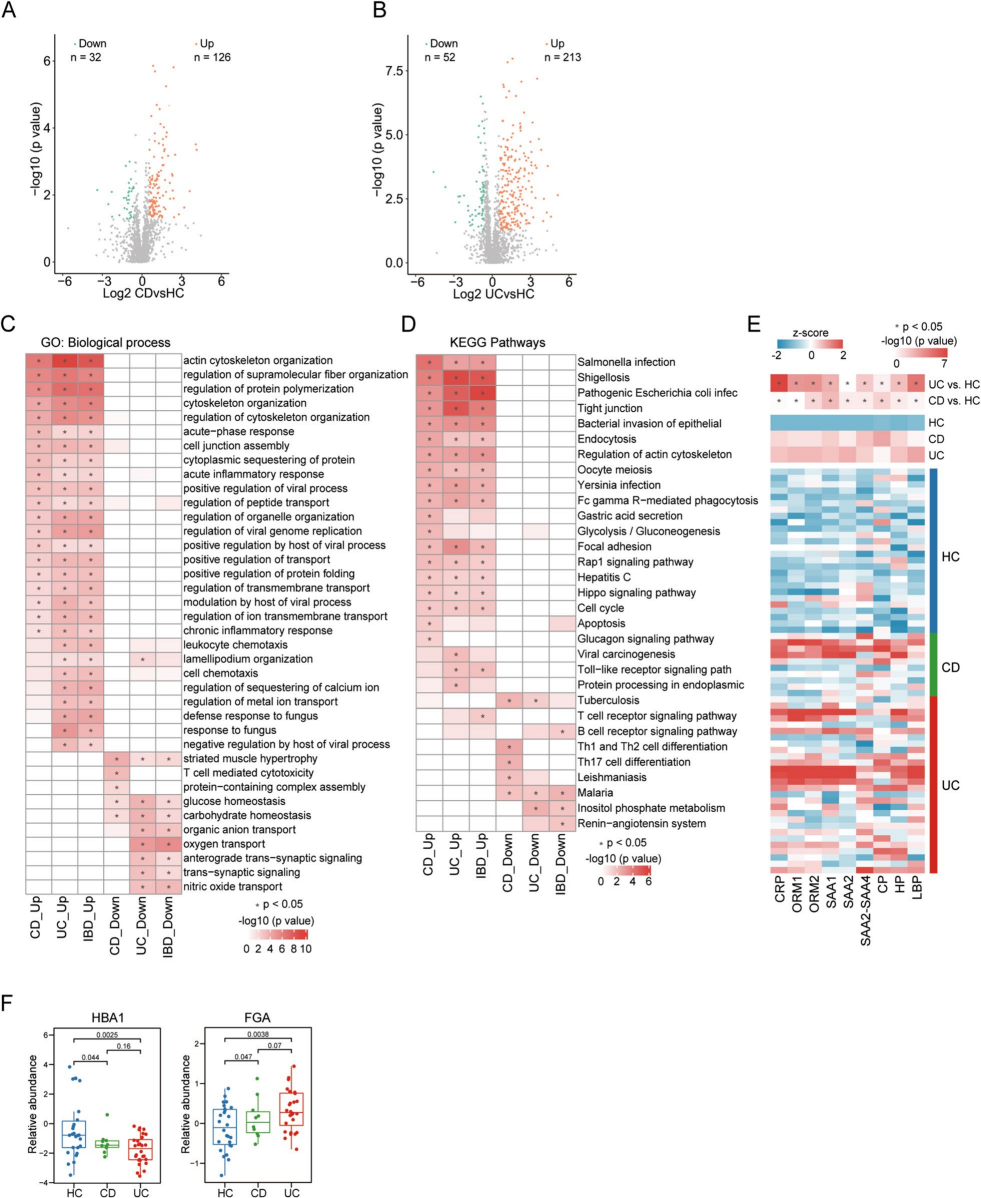
Differential expressed proteins (DEPs) in serum samples and GO and KEGG analysis for DEPs among UC, CD and HC groups. **A–B** Volcano plots showing the DEPs between CD as well as UC and HC. Orange and green plots represent up and down-regulated proteins. **C** Heatmap showing the GO biological process term enriched by the DEPs between IBD and HC. * indicates *P* < 0.05. **D** KEGG enrichments for these DEPs. * indicates *P* < 0.05. **E **Several acute-phase proteins are depicted by a z-score pseudocolor scale among groups. CRP: C reaction protein; ORM1: orosomucoid 1; ORM2: orosomucoid 2; SAA1: serum amyloid A1; SAA2: serum amyloid A2; SAA2-SAA4: serum amyloid A2-serum amyloid A4; CP: ceruloplasmin; HP: haptoglobin; LBP: lipopolysaccharide binding protein. The color of the bar indicates the z-score (blue, negative z-score; red, positive z-score). Comparation of aforementioned acute-phase proteins between UC as well as CD and HC are depicted by a -log10 (*P* value) pseudocolor scale. * indicates *P* < 0.05. **F** Box plots represent HBA1 and FGA abundance levels in the serum samples as markers for anemia and thromboembolic disease between CD as well as UC and HC. The inter-quartile ranges are shown. *P* < 0.05 is considered as significant (two-sided *t* test)

We obtained the DEPs in serum samples between IBD and HC groups using online PTM-BIO Shiny Tool. Figure 3A shows a volcano map representing DEPs between CD and HC groups. A total of 126 upregulated and 32 downregulated DEPs were identified. Moreover, 213 upregulated and 52 down-regulated DEPs were obtained between UC and HC groups (Fig. 3B). Then, these DEPs were subjected into eggNOG-mapper online software (v.2.0) for the GO functional annotation and KEGG pathway enrichment.

We annotated the DEPs in the serum using GO analysis to investigate the biological functional process involved in IBD (Fig. 3C). Some biological processes related to inflammatory response were identified among the 20 most upregulated biological processes for upregulated DEPs in IBD group compared with those in HC group, these responses included acute-phase response, acute inflammatory response, positive regulation of viral process, modulation by host of viral process, and chronic inflammatory response. Notably, several biological processes associated with cytoskeleton regulation can be observed among the 20 most upregulated biological processes in IBD. These processes involved actin cytoskeleton organization, regulation of cytoskeleton organization, regulation of supramolecular fiber organization, and cytoskeleton organization. In particular, most of the upregulated DEPs were associated with actin cytoskeleton organization and most of the downregulated DEPs were associated with striated muscle hypertrophy in CD group compared with those in HC group. In contrast to HC group, UC group had more upregulated DEPs enriching in actin cytoskeleton organization and more downregulated DEPs enriching in oxygen transport.

Through KEGG analysis (Fig. 3D), diverse KEGG pathways associated with intestinal microbial infection were found among the 20 most upregulated pathways in IBD, such as *Salmonella* infection, *Shigellosis*, pathogenic *Escherichia coli* infection, *Yersinia* infection, and Hepatitis C. Several significant upregulated pathways associated with bacterial pathogenesis were identified in IBD, such as tight junction, bacterial invasion of epithelial, and focal adhesion. Some pathways related to host response against infection in IBD were also found, including endocytosis, Fc gamma R-mediated phagocytosis, Rap1 signaling pathway, and Hippo signaling pathway. Notably, upregulated DEPs between CD group and HC group were significantly related to Salmonella infection, and downregulated DEPs were significantly associated with Th1 and Th2 cell differentiation, tuberculosis, Th17 cell differentiation, leishmaniasis, and malaria. Comparing UC group with HC group showed that Shigellosis was the most considerably upregulated pathway, and inositol phosphate metabolism was the most remarkably downregulated pathway.

Given that all eligible IBD patients were in active phase, diverse acute phase proteins, including C reaction protein (CRP), orosomucoid 1 (ORM1), orosomucoid 2 (ORM2), serum amyloid A1 (SAA1), serum amyloid A2 (SAA2), serum amyloid A2-serum amyloid A4 (SAA2-SAA4), ceruloplasmin (CP), haptoglobin (HP), and lipopolysaccharide binding protein (LBP), were further assessed (Fig. 3E). The results showed that notable elevations of the aforementioned acute-phase proteins were observed in CD and UC groups compared with those in HC group (all *P* < 0.05).

Anemia and thromboembolic events have been considered two common complications in IBD [36, 37]. We further analyzed HBA1 and FGA abundance levels in the peripheral blood as markers for anemia and thromboembolic disease, respectively (Fig. 3F). CD group (*P* = 0.044) and UC group (*P* = 0.0025) group had lower abundance levels of HBA1 than HC group. Meanwhile, elevated levels of FGA were observed in both UC group (*P* = 0.0038) and CD group (*P* = 0.047). These results correspond to the clinical manifestation of IBD.

### Altered gut microbiota between IBD patients and controls based on fecal metaproteome

**Fig. 4:**
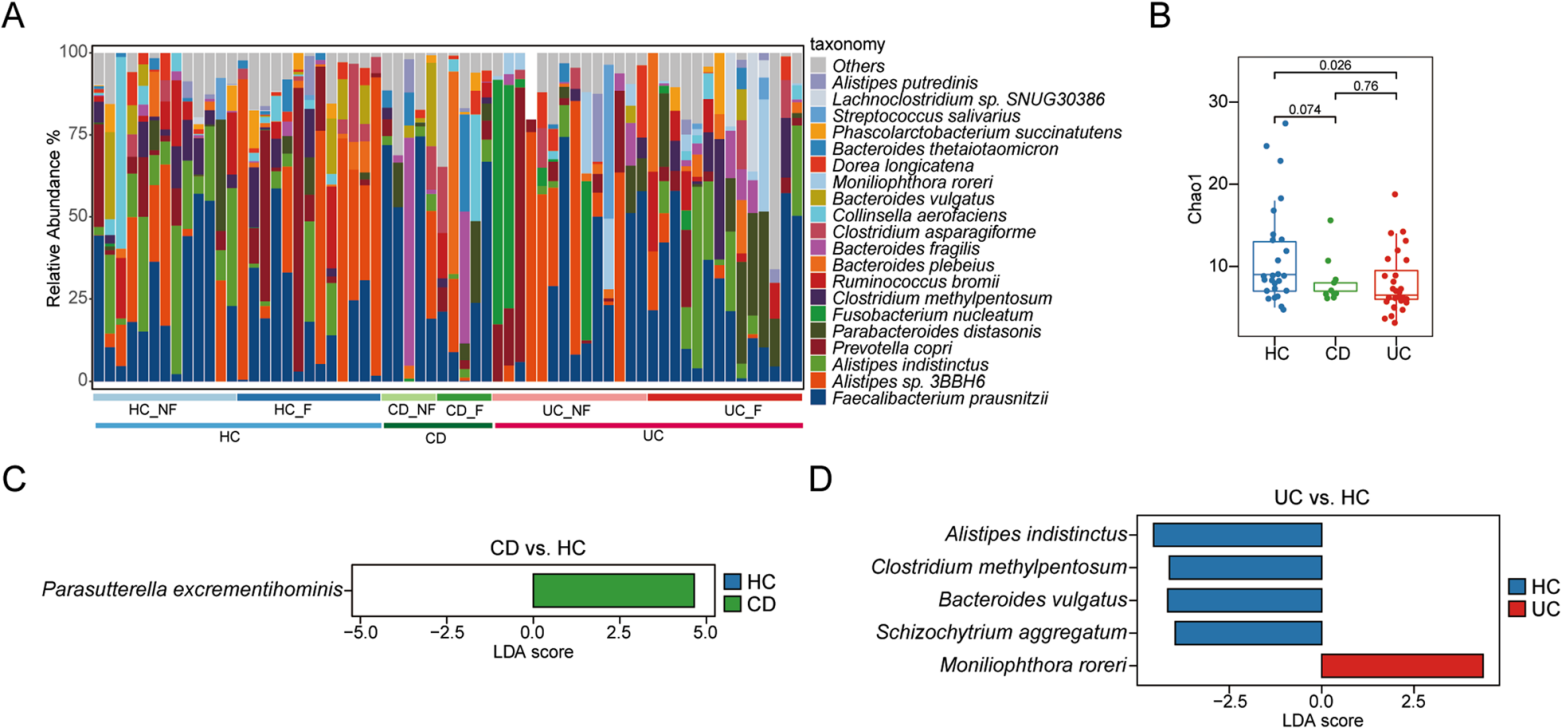
Fecal taxonomy, diversity, and gut microflora abundance analyses among UC, CD and HC groups. **A** Relative abundance of gut microflora species levels. **B** Alpha diversity was determined by *Chao1* richness. Box plot shows median (middle line) bounded by minimum, first quartile (lower border of the box), third quartile (upper border of the box) and maximum. *P* < 0.05 is considered as significant (two-sided *t* test). **C** Linear discriminant analysis (LDA) effect size (LEfSe) analysis between CD and HC. **D** LEfSe analysis between UC and HC

The strength of metaproteome is to identify previously unknown microbial flora based on protein profiles [38]. Herein, 66 species in the feces were identified. To investigate the difference in microbial community composition between IBD and HC groups, the top 20 most abundant species were exhibited (Fig. 4A). *Chao1* richness estimation was further used to assess alpha diversity among HC, CD, and UC groups. As shown in Fig. 4B, the *Chao1* index significantly decreased in UC group compared with that in HC group (*P* = 0.026). Conversely, CD group had a slight but an insignificant decrease in the *Chao1* index (*P* = 0.074). This result suggests that the gut microbiota compositions of UC patients were altered considerably.

We performed LDA effect size (LEfSe) on high-dimensional biomarkers to identify dominant microbiota species that significantly differed among groups. Among 66 species, *Phascolarctobacterium excrementihominis* was more enriched in CD group than in HC group (Fig. 4C). Comparing UC group with HC group showed that the former had a significant enrichment of *Moniliophthora roreri* (*M. roreri*) but dramatically decreased abundances of *A.indistinctus*, *Clostridium methylpentosum*, *Bacteroides vulgatus,* and *Schizochytrium aggregatum* (Fig. 4D). These findings suggest the alterations of gut microbial compositions in IBD patients.

### Impacts of overweight/obesity on IBD patients

**Fig. 5:**
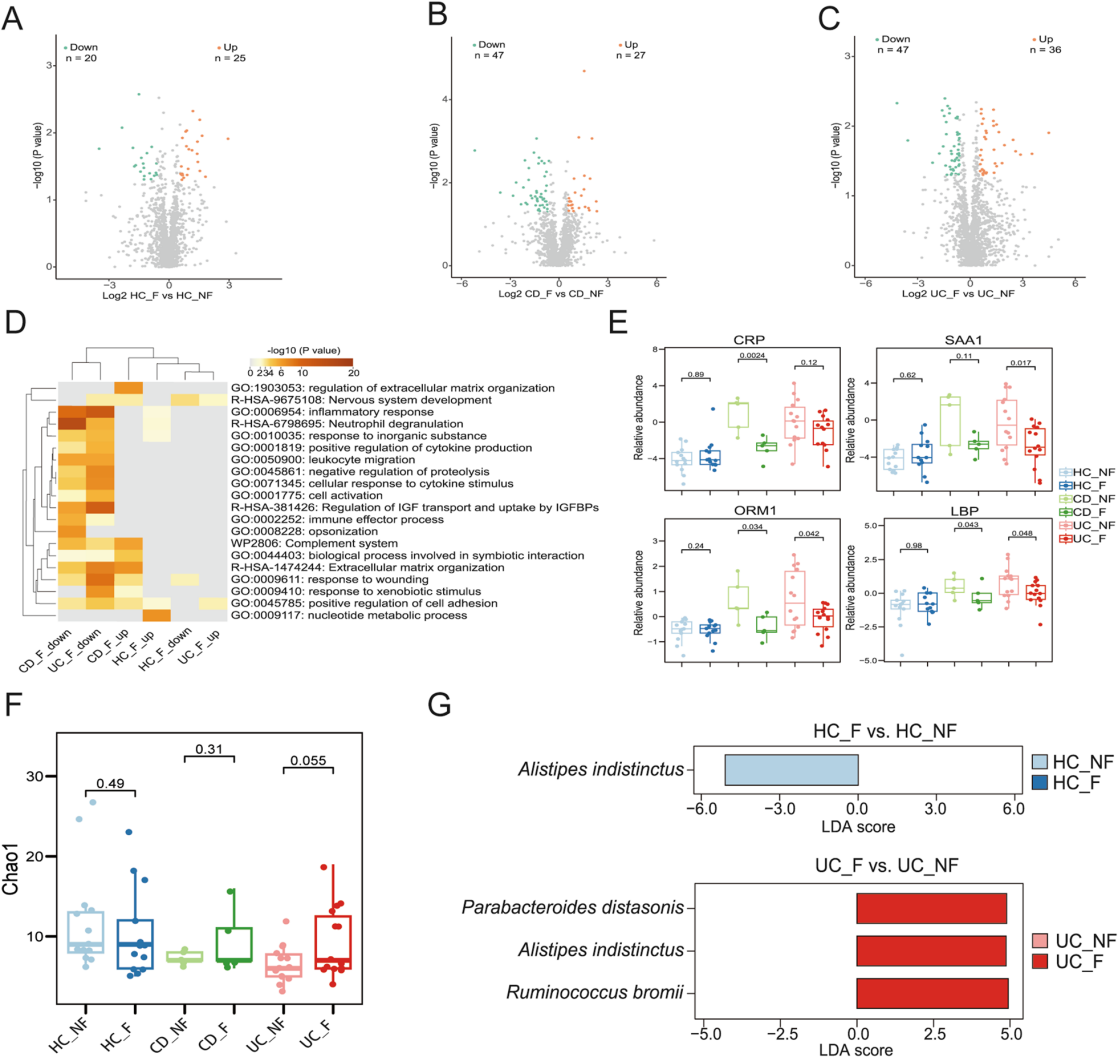
Serum proteome and fecal metaproteome analyses among UC_F, UC_NF, CD_F, CD_NF, HC_F, and HC_NF groups. **A** The impacts of obesity on the serum proteome in HC. Volcano plot showing the DEPs between HC_F and HC_NF. **B** The impacts of obesity on the serum proteome in CD patients. Volcano plot showing the DEPs between CD_F and CD_NF.** C** The impacts of obesity on the serum proteome in UC patients. Volcano plot showing the DEPs between CC_F and UC_NF. Orange and green plots represent up and down-regulated proteins. **D** Heatmap showing the GO terms and pathways enriched by altered serum proteins by overweight/obesity in the IBD patients using Metascape. Significant GO terms and pathways are shown. * indicates *P* < 0.05. **E** Comparison of CRP, SAA1, ORM1, and LBP levels in serum sample among six groups. **F** Alpha diversity was determined by *Chao1* richness. **G** LEfSe analysis for HC_F vs. HC_NF and UC_F vs. UC_NF. Box plot shows median (middle line) bounded by minimum, first quartile (lower border of the box), third quartile (upper border of the box) and maximum. *P* < 0.05 is considered as significant (two-sided *t* test)

We subsequently performed subgroup analyses for serum proteome and fecal metaproteome based on with or without overweigh/obesity to further analyze the impacts of overweight/obesity on IBD. Figure 5A–C show volcano plots representing 25, 27, and 36 upregulated DEPs and 20, 47, and 47 down-regulated DEPs in serum samples between HC_F and HC_NF, CD_F and CD_NF, and UC_F and UC_NF, respectively. These upregulated and downregulated DEPs were respectively annotated by GO and KEGG enrichment using the web tool “Metascape” (Fig. 5D). For HC_F versus HC_NF, up-regulated DEPs were enriched in nucleotide metabolic process and positive regulation of cell adhesion, while down-regulated DEPs were enriched in response to wounding. For CD_F versus CD_NF, extracellular matrix organization, complement system, and biological process involved in symbiotic interaction were enriched for up-regulated DEPs. The downregulated DEPs were mainly enriched in inflammatory response (GO:0006954), neutrophil degranulation (R-HSA-6798695), opsonization (GO:0008228), leukocyte migration (GO:0050900), and cellular response to cytokine stimulus (GO:0071345). Among them, neutrophil degranulation (R-HSA-6798695) was the most significantly enriched pathway. For UC_F versus UC_NF, up-regulated DEPs were enriched in positive regulation of cell adhesion and nervous system development. Several enriched GO terms for downregulated DEPs were related to inflammatory response, such as inflammatory response (GO:0006954), cellular response to cytokine stimulus (GO:0071345), leukocyte migration (GO:0050900), and positive regulation of cytokine production (GO:0001819). Regulation of IGF transport and uptake by IGFBPs (R-HSA-381426) and neutrophil degranulation (R-HSA-6798695) were the two most significantly enriched pathways. The obtained results indicate that the downregulated DEPs in CD_F versus CD_NF and UC_F versus UC_NF are mainly enriched in “inflammatory response”, that is, the CD_F and UC_F patients have a lower inflammatory response than their respective counterparts. Some classical inflammatory biomarkers, including CRP, SAA1, ORM1, and LBP, were determined to further confirm the notably lower inflammatory responses of IBD patients with overweight/obesity than those without overweight/obesity (Fig. 5E). The patients in CD_F group had significantly lower levels of CRP (*P* = 0.0024), ORM1 (*P* = 0.034), and LBP (*P* = 0.043) than those in CD_NF group. Meanwhile, dramatic reductions in SAA1 (*P* = 0.017), ORM1 (*P* = 0.042), and LBP (*P* = 0.048) were observed in UC_F group compared with those in UC_NF group. Collectively, these findings indicate that overweight/obesity may attenuate inflammatory response of IBD patients.

We also assessed the diversity of gut microbiome by *Chao1* index among HC, HC_F, UC_NF, UC_F, CD_NF, and CD_F groups (Fig. 5F). We found that the gut microflora among six groups fluctuated, and alpha diversity of intestinal flora in UC_F group and CD_F group were increased compared with that in UC_NF group and CD_NF group, but both were nonsignificant (*P* = 0.055, *P* = 0.31 respectively). We also implemented a LEfSe analysis to identify dominant microbiota species that differed significantly between the group with and without overweight/obesity (Fig. 5G). The LEfSe analysis showed that no significant microflora was found between CD_F and CD_NF in Fig. 5G. In contrast to those in UC_NF group, the patients in UC_F group had increased abundance of *P. distasonis*, *A. indistincus*, and *Ruminococcus bromii*. Interesting, *A. indistincus* was also significantly abundant in completely healthy subjects (HC_NF). Overall, we speculate that overweight/obesity may alleviate inflammatory response of UC patients by increasing probiotics abundance, but not CD patients.

## Discussion

Contrary to the previous beliefs of IBD as underweight and malnourished, increasing evidence shows that rates of obesity in IBD patients have increased in recent years [11]. The nature of any interaction between overweight/obesity and IBD remains poorly understood. Several common states related to overweight/obesity were identified through serum proteomics and fecal metaproteomic analyses of a carefully recruited cohort of IBD patients. First, the current preliminary findings based on serum proteomic demonstrate that overweight/obesity affects the protein profiles of UC and CD in the same ways. It is reflected by that the GO and KEGG enrichments related to the inflammatory response for downregulated DEPs of UC_F and CD_F are extremely similar. Moreover, metaproteomic-based analyses suggest that the differences in protein profiles between UC and CD groups may be attributed to the alterations of gut microbiome as affected by overweight/obesity.

Serum proteomic technology has been widely applied to the discovery of potential biomarkers for the diagnosis, severity and prognosis of IBD [39]. A recent large case–control study by Joana et al*.* identified a panel comprising 51 serum proteins that could assist in diagnosing CD 5 years in advance [40]. Another large European multicenter investigation demonstrated that a panel with 66 serum proteins had the potent ability to discriminate IBD from non-IBD controls and even predict the disease progression [21]. Similar results were also found in this study, in which 265 unique serum proteins were obtained in IBD patients compared with healthy subjects. Obesity is believed to represent a low-grade inflammatory state and lead to the discouraging therapeutic outcomes of IBD [41]. Some studies support weight loss as an obvious goal in the management and treatment of IBD [11]. However, we found in this study that most serum downregulated DEPs in IBD with overweight/obesity were dramatically enriched in the inflammatory responses (Fig. 5D), which suggests that overweight/obesity has the beneficial effects of alleviating inflammation in IBD patients. Our findings are congruent with those of Avegail et.al who conducted a 13-year retrospective study of patients with IBD, which supported that obesity should be an indicator of a less severe disease course of IBD [15].

Fecal metaproteomic, as an emerging multi-omics technology, is a powerful tool for identifying previously unknown microbial communities and understanding the function of the gut microbiota [42, 43]. A recent fecal metaproteomic report from Germany described negative associations between transcriptional regulatory protein RprY and human IgA from *Bacillus fragilis* and IBD. Positive relationships were also found between an increase in neutrophil extracellular traps and immune globulins and IBD [26]. Another published research of fecal metaproteomic showed that different IBD phenotypes differed in their taxonomic and functional landscapes [44]. In the present study, comparing UC and CD subgroup with controls showed that UC microbiomes were characterized by the increased activity of *M. roreri* and a significant reduction in relative abundance of *A. indistinctus*, *Clostridium methylpentosum*, *Bacteroides vulgatus*, and *Schizochytrium aggregatum*. Meanwhile, CD microbiomes were characterized by the increased abundance of *P. excrementihominis*. However, the results from a recent study by Carlos et al*.* are inconsistent with our findings [45], and these discrepancies may be attributable to different geographical locations, environments, and dietary components.

Substantial evidence links obesity to IBD, but few studies on taxonomic and functional profiles of microbial-derived proteins in the obese IBD patients based on fecal metaproteomic have been conducted. In this study, we found that the abundance of *P. distasonis* in UC with overweight/obesity was greater than that of the patients without overweight/obesity. Some previous investigations have suggested that *P. distasonis* could serve as a potential probiotic to improve human and animal digestive health [46–48]. *R. bromii* was also found to be more abundant in UC with overweight/obesity. As far back as a decade ago, Ze et al*.* proposed that *R. bromii* possessed excellent ability to degrade resistant starch particles in human colon [49]. The effect of *R. bromii* as a probiotic was further confirmed in subsequent investigations [50–52]. Interestingly, we also identified another important bacterial species that was highly abundant in the UC patients with overweight/obesity, that is, *A. indistinctus*. A higher bacterial abundance was observed in the completely healthy individuals than in those without overweight/obesity. *A. indistinctus* was first discovered and named by Fumiko et. al in 2009 [53]. Recently, an essential role has been ascribed to *A. indistinctus* in the maintenance of intestinal microflora balance and gastrointestinal health [54, 55]. Overall, we speculate that UC patients with overweight/obesity have alterations of gut microbial compositions, especially elevations of some probiotics. They induce beneficial protecting effects on the progression of IBD by mitigating the inflammatory response.

To the best of our knowledge, serum proteome and fecal metaproteome regarding overweight/obesity impacting on the development and progression of IBD have been rarely reported. Our study is one of the pioneer works in this field. Despite these promising findings, we acknowledge some limitations. First, since few IBD patients enrolled in our study had BMI greater than 28, the IBD patients with overweight and obesity were combined and the stratified analyses by overweight and obesity were not performed. Second, the present study was conducted in a single center with a small sample. Especially, only 10 patients with CD, that is, 5 CD_F and 5 CD_NF, were enrolled, which could partially explain no clear detectable differences in the fecal metaproteome between CD_F and CD_NF. Third, a validation study for the findings from UC_F compared with UC_NF was lacking. Therefore, further studies with multicenter and a large sample size for the extrapolation of our findings are warranted. We also did not perform fecal metagenomic analyses, and the identification of gut microbial communities relied mostly on public databases. Fecal metagenomic analysis thus merits consideration in subsequent studies. Finally, protein regulatory network associated with IBD in serum and fecal proteome was constructed, but no apparent correlation was observed and the results were not shown herein.

## Conclusion

Our multi-omics study is the first attempt to uncover the common states of serum protein dysregulation and microbiome dysbiosis in IBD patients with overweight/ obesity. Overweight/obesity can blunt inflammatory response of IBD patients by increasing probiotics abundance, especially in the UC patients. Despite the need for additional clinical validation and mechanistic analysis, our study emphasizes the importance of the further investigation of the potential association between IBD and overweight/obesity. This initiative may drive the clinical management of IBD patients with overweight/obesity.

## Data Availability

All sequences and associated metadata for the current study are available from the corresponding author upon reasonable request. On behalf of research collaboration and development, the patients and the ethics committee were allowed to share the data with the corresponding authors’ consideration.
